# A retrospective study of uropathogen and its antibiotic resistance among children with urinary tract infection from a single center in China

**DOI:** 10.1016/j.heliyon.2024.e31902

**Published:** 2024-05-23

**Authors:** Kaiping Zhang, Xiang Fang, Yin Zhang, Min Chao

**Affiliations:** Department of Urology, Anhui Provincial Children's Hospital/Children's Hospital of Fudan University (Affiliated Anhui Branch), Hefei, China

**Keywords:** Urinary tract infection, Complicated UTI, Multi-drug resistance, Antimicrobial susceptibility

## Abstract

Urinary tract infection (UTI) is a well-known bacterial infection posing serious health problem in children. A retrospective study was conducted to explore the uropathogen and its antibiotic resistance in children with UTI. Data of urine culture and antimicrobial susceptibility test was collected. Consequently, 840 children were included. The overall culture-positive UTI was 458 (54.52 %) with *Escherichia coli* 166 (36.24 %), followed by *Enterococcus faecalis* 59 (12.88 %), *Enterococcus faecium* 70 (15.28 %) and others. They were highly resistant to the most commonly used antibiotics. In 694 children with complicated UTI, there were 8 children with fungal infection. Multiple drug resistance (MDR) was recorded in 315 (80.98 %). The overall proportion of Extended Spectrum β-Lactamase (ESβL) production was 25 (6.43 %). In 146 children with simple UTI, MDR were also detected in 47 (77.05 %). There were 6 (9.84 %) positive for ESβL production. Our study found that complicated UTI was relatively common. *Escherichia coli* was the most prevalent isolate, followed by *Enterococcus faecium* and *Enterococcus faecalis*. These organisms were highly resistant to the most commonly used antibiotics. Relatively high prevalence of MDR and low ESβL-producing organisms were observed.

## 1 Introduction

Urinary tract infection (UTI) is an infectious disease in children. Approximately 8.4 % of girls and 1.7 % of boys under 7 years are diagnosed with UTI, and up to 30 % of children experience recurrent UTI by the age of 12 months [1,2]. The short-term symptoms usually contain fever, dysuria, and flank pain [3]. If not properly treated, it often develops recurrent UTI, kidney scar, and decline of renal function [4,5]. In contrast to adults, children are susceptible to UTI due to immune disorders, congenital anomalies of the genitourinary tract, and so on [6,7]. An indwelling catheter often introduces external pathogens into the bladder. Congenital abnormalities allow for the prolongation of bacteruria and decreasing the clearance of invading pathogens due to poor drainage.

In practice, it is vital to differentiate complicated UTI from simple UTI. The risk factors of complicated UTI include the functional or structural abnormalities of the genitourinary tract, foreign bodies, stones, and immunosuppression. It often leads to treatment failure and serious complications, which may require further evaluation [8]. UTI may be a clinical presentation for potential abnormality due to its poor drainage in children [9].In addition, some patients also have UTI with stone disease, and necessitate drainage [10,11]. Thus, the therapeutic goals of complicated UTI identify those potentially risk factors [12]. The abnormalities often cause inappropriate antibacterial treatment, which in turn increases antimicrobial resistance (AMR) [13].

Rapid administrations of empirical antibiotics are required before urine culture testing. The pathogen is detected and identified in urine which contributes to antibacterial treatment. There is an urgent need to identify bacterial strains and susceptibility to commonly used antimicrobial, as well as potentially risk factors in children.

## 2 Materials and methods

### 2.1 Study design

A retrospective analysis was performed at the department of Urology, Anhui Provincial Children's Hospital during the 6 years from January 2017 to December 2022. The general characteristics, the microorganism isolated, and the antimicrobial susceptibility profiles were collected. Written informed consents were collected from their parents.

### 2.2 Antimicrobial susceptibility testing

A midstream urine sample had taken from hospitalized children. Susceptibility testing was performed with the disk-diffusion method according to the criteria of National Committee for Clinical Laboratory Standards (NCCLs) [14]. The drugs included ampicillin (AMP), ampicillin-sulbactam (SAM), ciproﬂoxacin (CP), levofloxacin (LEV), nitrofurantoin (FM), piperacillin-tazobactam (TZP), cephazolin (CZO), cefotetan (CTT), ceftazidine (CAZ), ceftriaxone (CRO), imipenem (IMP), amikacin (AN), tobramycin (TOB), trimethoprim-sulfamethoxazole (TMP-STX), cefoxitin (CXT); imipenem (IPM), cefotaxime (CTX), aztreonam, ertapenem, gentamicin, cefepime, quinupristin, tigecycline, penicillin-G (PG), gentamycin (GM), erythromycin (E), linezolid (LZD), vancomycin (VA), tetracycline (TC), nitrofurantoin (FM).

### 2.3 Statistical analysis

A descriptive data was expressed as frequency and its percentage in our study such as the proportion of children, susceptibility patterns, MDR, and so on. In addition, the independent risk factor for culture positive UTI was tested by univariable logistic regression analyses. Data was analysed by SPSS statistical software package (version 16.0). *P*-value less than 0.05 was taken as statistically significant.

## 3 Results

As a result, there were 840 hospitalized patients with UTI. UTI was more common in male 569 (67.74 %) than female 271 (32.26 %). The age ranged from 1 month to 14 years. 694 (82.62 %) children were diagnosed with complicated UTI and the remaining 146 (17.38 %) children were simple UTI. The general characteristics were shown in Table 1.

**Table 1:** Clinical characteristics of study participants by diagnosis of UTI.

Variables		Total UTI (N = 840)	Complicated UTI (N = 694)	Simple UTI (N = 146)
Age(in years)	≤1y	458	403	55
	1-5y	204	149	55
	≥5y	178	142	36
Gender	Male	569	485	84
	Female	271	209	62
Mode of delivery	Vaginal delivery	469	386	83
	Caesarean delivery	371	308	63
Birth weight	Low (＜2500g).	34	25	9
	Normal (2500–4000g)	778	656	122
	High (＞4000g)	28	13	15
Feeding pattern	Breast feeding	496	398	98
	Artificial feeding	204	182	22
	Mixed feeding	140	114	26
Premature delivery (<37weeks)	Yes	34	28	6
	No	806	666	140
Primigravida	Yes	401	320	81
	No	439	374	65
Ureteral stent or catheter	Yes	213	213	0
	No	627	481	146
Clinical presentation	Yes	481	340	141
	No	359	354	5
History of surgery	Yes	294	294	0
	No	546	400	146

UTI, urinary tract infection.

The proportion of culture-positive UTI was 458 (54.52 %) including *Escherichia coli* 166 (36.24 %), *Enterococcus faecalis* 59 (12.88 %), *Enterococcus faecium* 70 (15.28 %), *Klebsiella pneumoniae* 33 (7.21 %), *Pseudomonas aeruginosa* 20 (4.37 %), *CoNS* 19 (4.15 %), *Enterobacter cloacae* 13 (2.84 %), *Citrobacter* 14 (3.06 %), *Proteus mirabilis* 12 (2.62 %) and others 52 (11.35 %). Among children with complicated UTI, 30 (4.32 %) had urinary stones and 664 (95.68 %) had urogenital abnormalities. The proportion of culture-positive UTI was 397 (57.20 %). Gram-negative bacteria mainly included *Escherichia coli* 121 (30.48 %), *Klebsiella pneumoniae* 32(8.06 %), *Pseudomonas aeruginosa* 20 (5.04 %), *Enterobacter cloacae* 11 (2.77 %) and *Citrobacter* 13 (3.28 %). The isolated Gram-positive bacteria mainly contained *Enterococcus faecalis* 58 (14.61 %) and *Enterococcus faecium* 66 (16.62 %). In addition, there were 146 children with simple UTI. We detected that the proportion of culture-positive UTI was 61/146 (41.78 %). The predominant bacteria was *Escherichia coli* (73.77 %, 45/61) followed by *Enterococcus faecium and CoNS* (each 6.56 %) (Table 2).

**Table 2:** Bacterial profile isolated from urine in both simple and complicated UTI.

Isolates	Total UTI (N = 458)	Simple UTI (N = 61)	Complicated UTI (N = 397)
***Escherichia coli***	166	45	121
***Enterococcus faecium***	70	4	66
***Enterococcus faecalis***	59	1	58
***Klebsiella pneumoniae***	33	1	32
***Pseudomonas aeruginosa***	20	0	20
***Citrobacter***	14	1	13
***CoNS***	19	4	15
***Proteus mirabilis***	12	2	10
***Enterobacter cloacae***	13	2	11
***Others***	52	1	51

UTI, urinary tract infection.

The antimicrobial susceptibility always varied. For children with complicated UTI, Gram-negative bacteria mainly included *Escherichia coli*, *Klebsiella pneumoniae*, *Pseudomonas aeruginosa*, *Enterobacter cloacae* and *Citrobacter*. We found that *Escherichia coli* isolates were highly sensitive to amikacin (95.87 %), ertapenem (91.74 %), nitrofurantoin (92.56 %), imipenem (90.08 %), and cefotetan (87.60 %) and high rates of resistance were also detected to ampicillin (93.39 %), cephazolin (76.03 %), ceftriaxone (73.55 %), trimethoprim-sulfamethoxazole (63.64 %) and aztreonam (64.46 %). *Klebsiella pneumoniae* isolates showed highly sensitive to ertapenem (96.87 %), amikacin (96.87 %), imipenem (93.75 %) and piperacillin-tazobactam (90.62 %). However, they were highly resistant to ampicillin (96.87 %), cephazolin (75.00 %), ceftazidine (62.50 %), ceftriaxone (62.50 %), aztreonam (62.50 %) and ampicillin-sulbactam (59.37 %) (Table 3). In addition, the Gram-positive bacteria of complicated UTI mainly contained *Enterococcus faecalis* and *Enterococcus faecium. Enterococcus faecalis* were highly sensitive to vancomycin, penicillin-G, tigecycline, linezolid and nitrofurantoin (100 %, 94.83 %, 88.48 %, 87.93 %, 86.20, respectively) and highly resistant to tetracycline (86.21 %), quinupristin (84.48 %), erythromycin (75.86 %). Likewise, *Enterococcus faecium* were highly sensitive to vancomycin, linezolid, tigecycline, quinupristin (98.48 %, 95.45 %, 84.85 %, 83.33 %), while highly resistant to ampicillin (93.94 %), erythromycin (89.39 %), penicillin-G (84.85 %), ciproﬂoxacin (77.27 %), levofloxacin (68.18 %) and tetracycline (66.67 %). For children diagnosed with simple UTI, *Escherichia coli* were highly sensitive to ertapenem (100 %), imipenem (100 %), and cefotetan (100 %), amikacin (95.56 %) and nitrofurantoin (91.11 %) and high rates of resistance were detected to ampicillin (91.11 %), trimethoprim-sulfamethoxazole (64.44 %), cephazolin (62.22 %) and ceftriaxone (53.33 %) (Table 4).

**Table 3:** Antimicrobial susceptibility patterns of Gram-negative bacteria from complicated UTI samples.

Isolates	Pattern	Amp	SAM	CP	LEV	FM	TZP	CZO	CTT	CAZ	CRO	Aztreonam	Ertapenem	IMP	AN	Gentamicin	TOB	TMP-STX	Cefepime
*Escherichia coli* (N = 121)	S	8 (6.61)	27 (22.31)	49 (40.50)	32 (26.45)	112 (92.56)	102 (84.30)	29 (23.97)	106 (87.60)	56 (46.28)	32 (26.45)	43 (35.54)	111 (91.74)	109 (90.08)	116 (95.87)	73 (60.33)	71 (58.68)	42 (34.71)	47 (38.84)
	I	0 (0.00)	22 (18.18)	5 (4.13)	25 (20.66)	6 (4.96)	5 (4.13)	0 (0.00)	1 (0.83)	6 (4.96)	0 (0.00)	0 (0.00)	0 (0.00)	3 (2.48)	1 (0.83)	3 (2.48)	27 (22.31)	2 (1.65)	10 (8.27)
	R	113 (93.39)	72 (59.51)	67 (55.37)	64 (52.89)	3 (2.48)	14 (11.57)	92 (76.03)	14 (11.57)	59 (48.76)	89 (73.55)	78 (64.46)	10 (8.26)	9 (7.44)	4 (3.30)	45 (37.19)	23 (19.01)	77 (63.64)	64 (52.89)
*Klebsiella pneumoniae* (N = 32)	S	0 (0.00)	10 (31.25)	20 (62.50)	20 (62.50)	14 (43.75)	29 (90.62)	8 (25.00)	26 (81.25)	10 (31.25)	11 (34.37)	12 (37.50)	31 (96.87)	30 (93.75)	31 (96.87)	27 (84.38)	27 (84.37)	15 (46.87)	19(59.38)
	I	1 (3.13)	3 (9.38)	3 (9.38)	6 (18.75)	8 (25.00)	2 (6.25)	0 (0.00)	0 (0.00)	2 (6.25)	1 (3.13)	0 (0.00)	0 (0.00)	0 (0.00)	1 (3.13)	0 (0.00)	3 (9.38)	0 (0.00)	0(0.00)
	R	31 (96.87)	19 (59.37)	9 (28.12)	6 (18.75)	10 (31.25)	1 (3.13)	24 (75.00)	6 (18.75)	20 (62.50)	20 (62.50)	20 (62.50)	1 (3.13)	2 (6.25)	0 (0.00)	5 (15.62)	2 (6.25)	17 (53.13)	13 (40.62)
*Pseudomonas aeruginosa* (N = 20)	S	1 (5.00)	0 (0.00)	19 (95.00)	19 (95.00)	3 (15.00)	20 (100)	2 (10.00)	/	19 (95.00)	2 (10.00)	12 (60.00)	13 (65.00)	5 (25.00)	20 (100)	20 (100)	20 (100)	/	10 (50.00)
	I	3 (15.00)	2 (10.00)	0 (0.00)	0 (0.00)	3 (15.00)	0 (0.00)	0 (0.00)	/	0 (0.00)	2 (10.00)	2 (10.00)	0 (0.00)	3 (15.00)	0 (0.00)	0 (0.00)	0 (0.00)	/	0 (0.00)
	R	16 (80.00)	18 (90.00)	1 (5.00)	1 (5.00)	14 (70.00)	0 (0.00)	18 (90.00)	/	1 (5.00)	16 (80.00)	6 (30.00)	7 (35.00)	12 (60.00)	0 (0.00)	0 (0.00)	0 (0.00)	/	10 (50.00)
*Enterobacter cloacae* (N = 11)	S	2 (18.18)	3 (27.27)	11 (100)	11 (100)	7 (63.64)	8 (72.73)	0 (0.00)	1 (9.09)	7 (63.64)	7 (63.64)	7 (63.64)	10 (90.91)	7 (63.64)	11 (100)	11 (100)	11 (100)	11 (100)	10 (90.91)
	I	2 (18.18)	3 (27.27)	0 (0.00)	0 (0.00)	4 (36.36)	1 (9.09)	0 (0.00)	2 (18.18)	0 (0.00)	0 (0.00)	1 (9.09)	0 (0.00)	2 (18.18)	0 (0.00)	0 (0.00)	0 (0.00)	0 (0.00)	0 (0.00)
	R	7 (63.64)	5 (45.46)	0 (0.00)	0 (0.00)	0 (0.00)	2 (18.18)	11 (100)	8 (72.73)	4 (36.36)	4 (36.36)	3 (27.27)	1 (9.09)	2 (18.18)	0 (0.00)	0 (0.00)	0 (0.00)	0 (0.00)	2 (18.18)
*Citrobacter* （N = 13)	S	0 (0.00)	1 (7.69)	5 (38.46)	5 (38.46)	7 (53.84)	6 (46.15)	1 (7.69)	/	5 (38.46)	2 (15.38)	2 (15.38)	10 (76.92)	9 (69.23)	13 (100)	6 (46.15)	7 (53.85)	2 (15.39)	2 (15.39)
	I	0 (0.00)	0 (0.00)	1 (7.69)	1 (7.69)	3 (23.08)	0 (0.00)	1 (7.69)	/	0 (0.00)	0 (0.00)	2 (15.38)	1 (7.69)	1 (7.69)	0 (0.00)	1 (7.70)	0 (0.00)	0 (0.00)	0 (0.00)
	R	13 (100)	12 (92.31)	7 (53.85)	7 (53.85)	3 (23.08)	7 (53.85)	11 (84.62)	/	8 (61.54)	11 (84.62)	9 (69.24)	2 (15.39)	3 (23.68)	0 (0.00)	6 (46.15)	6 (46.15)	11 (84.61)	11 (84.61)

Amp, Ampicillin. SAM, Ampicillin and Sulbactam. CP, Ciproﬂoxacin. LEV, Levofloxacin. FM, Nitrofurantoin. TZP, Piperacillin and Tazobactam. CZO, Cephazolin. CTT, Cefotetan. CAZ, Ceftazidine. CRO, Ceftriaxone. IMP, Imipenem. AN, Amikacin. TOB, Tobramycin. TMP-STX, Trimethoprim-sulfamethoxazole. CXT, Cefoxitin. IPM, Imipenem. CTX, Cefotaxime. CoNS, Coagulase negative staphylococcus. S, I, R, sensitive, intermediate, resistant.

**Table 4:** Antimicrobial susceptibility patterns of Gram-positive bacteria from simple UTI samples.

Isolates	Pattern	PG	Amp	GM	CP	LEV	E	Quinupristin	LZD	VA	TC	Tigecycline	FM
***Enterococcus faecalis* (N=58)**	S	55 (94.83)	47 (81.03)	33 (56.90)	36 (62.07)	46 (79.31)	1 (1.72)	6 (10.35)	51 (87.93)	58 (100.00)	8 (13.79)	49 (88.48)	50 (86.20)
	I	0 (0.00)	0 (0.00)	0 (0.00)	9 (15.52)	5 (8.62)	13 (22.42)	3 (5.17)	3 (5.17)	0 (0.00)	0 (0.00)	2 (3.45)	4 (6.90)
	R	3 (5.17)	11 (18.97)	25 (43.10)	13 (22.41)	7 (12.07)	44 (75.86)	49 (84.48)	4 (6.90)	0 (0.00)	50 (86.21)	7 (12.07)	4 (6.90)
***Enterococcus faecium* (N=66)**	S	8 (12.12)	4 (6.06)	27 (40.91)	5 (7.58)	8 (12.12)	0 (0.00)	55 (83.33)	63 (95.45)	65 (98.48)	22 (33.33)	56 (84.85)	30 (45.45)
	I	2 (3.03)	0 (0.00)	0 (0.00)	10 (15.15)	13 (19.70)	7 (10.61)	5 (7.58)	3 (4.55)	1 (1.52)	0 (0.00)	3 (4.55)	13 (19.70)
	R	56 (84.85)	62 (93.94)	39 (59.09)	51 (77.27)	45 (68.18)	59 (89.39)	6 (9.09)	0 (0.00)	0 (0.00)	44 (66.67)	7 (10.60)	23 (34.85)
***CoNS* (N=15)**	S	0 (0.00)	0 (0.00)	6 (40.00)	5 (33.33)	6 (40.00)	1 (6.67)	11 (73.33)	15 (100.00)	15 (100.00)	9 (60.00)	7 (46.67)	12 (80.00)
	I	1 (6.67)	0 (0.00)	0 (0.00)	1 (6.67)	2 (13.33)	3 (20.00)	1 (6.67)	0 (0.00)	0 (0.00)	0 (0.00)	1 (6.66)	1 (6.67)
	R	14 (93.33)	15 (100.00)	9 (60.00)	9 (60.00)	7 (46.67)	11 (73.33)	3 (20.00)	0 (0.00)	0 (0.00)	6 (40.00)	7 (46.67)	2 (13.33)

PG, penicillin-G. Amp, Ampicillin. GM, Gentamycin. CP, Ciproﬂoxacin. LEV, Levofloxacin. E, erythromycin. LZD, Linezolid. VA, Vancomycin. TC, Tetracycline. FM, Nitrofurantoin. S, I, R, sensitive, intermediate, resistant.

There were 458 culture-positive UTIs. Of these, 450 patients was bacterial infection. MDR were recorded in 362 (80.44 %). Among 397 culture-positive complicated UTI, there were 8 children with fungal infection and 389 children diagnosing bacterial isolates. MDR were recorded in 315 (80.98 %). The presence of ESβL was found in *Klebsiella pneumoniae, Klebsiella oxytoca* and *Escherichia coli* isolates. The overall proportion of ESβL production was 25 (6.43 %). Specifically, there were 21 *Escherichia coli*, 3 *Klebsiella pneumoniae* and 1 *Klebsiella oxytoca* positive for ESβL production (Table 5). MDR of simple UTI were also detected in 47 (77.05 %). There were only 6 (9.84 %) *Escherichia coli* positive for ESβL production. Univariable logistic regression analyses showed significant associations between prevalence of culture positive UTI and age, UTI type (*P* < 0.05) (Table 6). We found that there were 305, 153 culture-positive UTIs in male and female, respectively. No statistical difference was detected between gender and culture positive UTI (*P* > 0.05). All the other factors were also not statistically significant.

**Table 5:** Multi drug resistance pattern and ESβL production of bacterial isolates from children with complicated UTI.

Isolates	N	ESβL, N(%)	MDR, N(%)	R0	R1	R2	R3	R4	R5	R6	≥R7
*Escherichia coli*	121	21 (17.36)	107 (88.43)	4	4	6	9	7	6	12	73
*Klebsiella pneumoniae*	32	3 (9.38)	22 (68.75)	1	7	2	0	1	1	3	17
*Pseudomonas aeruginosa*	20	0 (0.00)	4 (20.00)	1	12	3	0	0	0	1	3
*Enterococcus faecalis*	58	0 (0.00)	47 (81.03)	1	3	7	13	16	10	4	4
*Enterococcus faecium*	66	0 (0.00)	65 (98.48)	0	0	1	1	8	9	12	35
*Enterobacter cloacae*	11	0 (0.00)	7 (63.64)	0	0	4	1	1	2	0	3
*Citrobacter*	13	0 (0.00)	8 (61.54)	2	0	3	1	1	1	0	5
*CoNS*	15	0 (0.00)	14 (93.33)	0	1	0	1	1	0	0	12
*Proteus mirabilis*	10	0 (0.00)	6 (60.00)	0	3	1	0	1	0	2	3
*Klebsiella oxytoca*	8	0 (0.00)	4 (50.00)	0	1	3	0	0	0	1	3
*Enterobacter aerogenes*	7	0 (0.00)	7 (100)	0	0	0	0	1	1	0	5
Others	28	1 (3.57)	24 (85.71)	1	2	1	4	2	3	8	7
Total	389	25 (6.43)	315 (80.98)	10	33	31	30	39	33	43	170

ESβL, Extended Spectrum β-Lactamase. MDR, multiple drug resistance. R1, resistance to one drugs. R2, resistance to two drugs. R3, resistance to three drugs. R4, resistance to four drugs. R5, resistance to five drugs. R6, resistance to six drugs. R7, resistance to seven drugs.

**Table 6:** Univariablee logistic regression analyses for factors associated with culture-positive UTI among children with UTI.

Variables		N (%)	Culture-positive	OR (95%CI)	*P* value
Age(in years)	≤1y	458 (54.52)	275	Ref	
	1-5y	204 (24.29)	101	0.653 (0.468–0.910)	0.012
	≥5y	178 (21.19)	82	0.568 (0.401–0.806)	0.001
Gender	Male	569 (67.74)	305	Ref	
	Female	271 (32.26)	153	1.122 (0.839–1.502)	0.437
UTI type	Simple UTI	146 (17.38)	61	Ref	
	Complicated UTI	694 (82.62)	397	1.863 (1.297–2.674)	0.001
Ureteral stent or catheter	Yes	213 (25.36)	113	Ref	
	No	627 (74.64)	345	1.083 (0.793–1.479)	0.617
Clinical presentation	Yes	481 (57.26)	250	Ref	
	No	359 (42.74)	208	1.273 (0.966–1.677)	0.086
History of surgery	Yes	294 (35.00)	154	Ref	
	No	546 (65.00)	304	1.142 (0.859–1.518)	0.360

Ref, reference; OR, odds ratio; UTI, Urinary tract infection.

## 4 Discussion

UTIs are found in nearly 0.7 % of girls and 2.7 % of boys by the age of 1 year [15]. Moreover, about 5 % of febrile girls and 20 % of febrile boys under two months are diagnosed with UTI [16]. UTIs are more common in premature infants than term infants during neonatal period [17]. There are 30 % children within 6 months and even up to 50 % school-age girls with recurrent UTI [18,19]. The high prevalence of UTI and fear of its recurrence often lead to irrational use of antibiotics. Empirical treatment is also prevalent in primary hospital [20,21].

Complicated UTIs are defined as those infections causing severe complications, such as urosepsis, renal scarring, and end-stage renal disease. Those risk factors in children are stones, indwelling catheters, and abnormalities of the genitourinary tract [22]. Anatomical or functional abnormalities allow for the prolongation of bacteruria and decreasing the clearance of invading pathogens due to poor drainage [23]. Children with posterior urethral valves (PUV) have increased risks of UTI for voiding dysfunction, elevating bladder pressure stemming and ongoing renal damage. The continued bladder pressure from urine stasis or obstruction can causes vesicoureteric reflux (VUR) and increases renal function damage by an ascending infection [24]. Approximately 20 % of neonatal cases with VUR were diagnosed with UTI and then led to renal scars [25,26]. In addition, UTI remains the first cause of morbidity and the second cause of mortality in patients with neurogenic bladder due to high bladder pressure, detrusor exyernal sphincter dyssynergia and post-void residual [27]. These children often require clean intermittent catheterisation, which further increase the UTI risk [28,29]. It is important to identify these abnormalities early because they may serve as a reservoir for bacterial growth or recurrent infections.

UTI is mostly caused by Gram-negative bacillus because of its unique structure which promotes the epithelial cells attachment and prevents bacteria from urinary lavage [30]. *E. coli* is the most predominant bacterium isolated from urine [30]. Other common Gram-negative pathogens include *Klebsilla pneumoniae*, *Proteus mirabilis*, *Enterobacter*, *Citrobacter*, and *P. aeruginosa*. Besides, Streptococcus species and Staphylococcus species are the frequent Gram-positive bacteria. In the present study, we also found the similar results.

In the group of complicated UTI, we found that Gram-negative isolates were shown high sensitivity to amikacin, ertapenem, nitrofurantoin, imipenem, and cefotetan, and highly resistant to ampicillin, cephazolin, ceftriaxone, trimethoprim-sulfamethoxazole and aztreonam. Gram positive bacteria showed high sensitivity to vancomycin, penicillin-G, tigecycline, linezolid and nitrofurantoin and highly resident to tetracycline, quinupristin and erythromycin. For simple UTI patients, the most common uropathogen was *Escherichia coli.* It was highly sensitive to ertapenem, imipenem, cefotetan, amikacin and nitrofurantoin and high rates of resistant were also observed to ampicillin, trimethoprim-sulfamethoxazole, cephazolin and ceftriaxone. Majority of the isolates were highly resistant to the commonly prescribed antibiotics. Thus, empirical treatment should be minimized as sensitivity always varies for each organism over time.

For empirical treatments worldwide, AMR is a major problem, especially in regions with limited resources. Presence of MDR organisms was often detected. ESβL producing strains can easily hydrolyze β-lactam agents that the bacteria resist antibiotics. These organisms also carry genes encoding resistance to antibiotics. The presence of ESβL was often found in the members of Enterobacteriaceae family, particularly *Klebsiella pneumoniae* and *Escherichia coli.* In the present study, MDR was relatively high in both groups. Complicated UTI organisms showed a relatively higher MDR. In addition, the prevalence of ESβL in children was relative low.

There are some limitations. Firstly, this study was performed in a single center. Secondly, our study is a retrospective study. Thirdly, some asymptomatic patients in clinic were not included. Eventually, contamination of voided specimens from infants is a problem to be solved.

## 5 Conclusion

Our study revealed a higher prevalence of complicated UTI. The majority of the isolates was Gram-negative bacteria. *Escherichia coli* was the most prevalent isolate followed by *Enterococcus faecium* and *Enterococcus faecalis*. These organisms were highly resistant to the most commonly used antibiotics. Relatively high MDR and low ESβL-producing organisms were commonly observed in both groups. Urine culture for antimicrobial susceptibility test is vital for appropriate management of UTI.

## Compliance with ethics guidelines

Kaiping Zhang, Xiang Fang, Yin Zhang and Min Chao declare no conflicts of interest. This manuscript is a retrospective study and does not involve a research protocol requiring approval by the relevant institutional review board or ethics committee.

## Additional information

No additional information is available for this paper.

## Data availability statement

All data was available in the article.

## CRediT authorship contribution statement

**Kaiping Zhang:** Writing – review & editing, Writing – original draft, Visualization, Data curation, Conceptualization. **Xiang Fang:** Writing – original draft, Data curation, Conceptualization. **Yin Zhang:** Writing – original draft, Visualization. **Min Chao:** Writing – review & editing, Conceptualization.

## Declaration of competing interest

The authors declare that they have no known competing financial interests or personal relationships that could have appeared to influence the work reported in this paper.
